# Salespeople in the Surgical Suite: Relationships between Surgeons and Medical Device Representatives

**DOI:** 10.1371/journal.pone.0158510

**Published:** 2016-08-03

**Authors:** Bonnie O’Connor, Fran Pollner, Adriane Fugh-Berman

**Affiliations:** 1 Alpert School of Medicine, Brown University, Providence, Rhode Island, United States of America; 2 Retired Journalist, Takoma Park, Maryland, United States of America; 3 Georgetown University Medical Center, Washington, District of Columbia, United States of America; Mayo Clinic Minnesota, UNITED STATES

## Abstract

**Background:**

Industry payments to surgeons have received public attention, but little is known about the relationships between surgeons and medical device representatives. Medical device representatives ("device reps") have become an integral part of operating room personnel. The effect of their presence on patient care deserves discussion.

**Study Design:**

We conducted a qualitative, ethnographic study to explore relationships between surgeons and medical device representatives, and characterize industry involvement in the training of surgeons. We used group and individual open-ended interviews to gain insight into the beliefs, values, and perspectives of surgeons and device reps. We conducted two focus groups, one with ear, nose, and throat surgeons, and one with hospital-based attending orthopedic surgeons. We also conducted individual interviews with three former or current medical device representatives, a director of a surgical residency program at an academic medical center, and a medical assistant for a multi-physician orthopedic practice.

**Results:**

While surgeons view themselves as indisputably in charge, device reps work hard to make themselves unobtrusively indispensable in order to establish and maintain influence, and to imbue the products they provide with personalized services that foster a surgeon's loyalty to the reps and their companies. Surgeons view industry-funded training opportunities as a necessary service. Device reps and some surgeons believe that reps benefit patient care, by increasing efficiency and mitigating deficiencies among operating room personnel (including the surgeons themselves).

**Conclusions:**

Our study raises ethical questions about the reliance of surgeons on device reps and device companies for education and surgical assistance and practical concerns regarding existing levels of competence among OR personnel.

## Introduction

In recent decades, medical device representatives (“device reps”) have quietly become an integral part of operating room personnel. While relationships with pharmaceutical sales representatives have been shown to influence physician prescribing behavior [1, 2] and reduce physicians awareness of adverse effects of drugs,[3] there has been little public discussion of the relationships between surgeons and device reps and how these relationships affect perceptions of specific products and affect patient care.

Although hospitals pay for joint implants, cardiac stents, and other implantable surgical devices, physicians largely choose which products to use—up to 61% of what is spent on supplies is for these so-called physician preference items.[4] The influence a device rep may exert on this choice is reflected in one study showing an association between the presence of sales reps in the cardiac catheterization laboratory over the course of one year with increased use of the respective companies’ drug-eluting stents, resulting in higher procedural costs.[5]

Device rep input into a surgeon’s implantable device choices is especially relevant in the context of the FDA 510(k) device approval category, which allows devices deemed to have “substantial equivalence” to already marketed products to be implanted into humans without clinical testing. 510(k) approval enables the manufacturer to circumvent the more stringent premarket proofs of safety and efficacy required of new devices. Lack of such proofs, coupled with aggressive marketing and early adoption of 510(k) devices, can sometimes lead to unexpected and dramatic failures, for example, when certain metal-on-metal total hip replacement systems failed, causing chronic pain and disability and the need for revision surgery. These devices were removed from the market—after 500,000 had been implanted.[6]

The financial connections between the device industry and surgeons are widespread and substantial and may contribute to a surgeon’s reliance on industry product claims. The literature abounds with reports documenting the extent to which surgeons receive payments from industry—more than half of all surgeons, with orthopedists leading with respect both to the number receiving payments and the amount of money received.[7–10]

The question of whether payments affect therapeutic choices is important. Orthopedic surgeons tend to use one vendor for most of their implants and maintain brand loyalty over an extended time.[11] In 2007, five companies that sell 95% of the hip and knee implants in the United States disclosed payments of more than $198 million to 939 orthopedic surgeons as part of a settlement with the U.S. Department of Justice.[12] Total payments to orthopedic surgeons dropped 60% between 2007 and 2008, and one analysis of hospital discharge data from Florida, New York, and urban Pennsylvania found that loss of payments led physicians to switch 7% of their device utilization from sponsoring firms to competing firms.[13]

A 2007 survey of 11 hospitals in New York City found that surgeons (including ob-gyns) were significantly more likely than non-surgeons to approve of industry funding of residency programs (76% vs. 56%), and were more likely to approve of industry-funded meals (83% vs. 69%), travel expenses (68% vs. 49%,) and payments for attending lectures (60% vs. 44%).[14]

Surgeons ally more closely with device representatives than with hospital representatives.[14] Indeed, this dynamic was decried by a hospital vice-president as an “incestuous bucket of worms” that challenges hospital attempts to standardize the product selection process in order to reduce the costs of physician preference items.[4] Device reps play an active role in surgery. An anonymous web-based survey of 43 healthcare industry representatives found that 88% (38 of 43) provided verbal instruction to a surgical team during a surgery, and 21% (nine of 43) had direct physical contact with a surgical team or patient during a surgery.[15] More than a third (16 of 43) had participated in a surgery in which they felt that their involvement was excessive.

## Methods

We conducted a qualitative study to explore relationships between surgeons and medical device reps, and to characterize industry involvement in the training of surgeons. We chose an ethnographic approach, using group and individual open-ended interviews, as uniquely suited to gaining insight into the beliefs, values, opinions, and perspectives of both individuals and identity-groups. The study was funded by a grant from Georgetown University’s Engaged Ethics Initiative for collaborative work on complex moral problems and approved by the Georgetown University Medical Center IRB.

As approved by the IRB, oral informed consent was obtained from all participants and included in the audio recordings and transcripts. We summarized aloud a study description and consent form (provided in hard copy to any participants who wished to have it) and then requested individual oral consent “on the record and for the record.” All participants agreed to participate, and oral consent took the form of conversational terms of agreement (such as “sure,” “no problem,” etc.).

Between 2013 and 2014, we conducted two focus groups, with a total of 14 surgeons at two academic medical centers, and five individual interviews, constituting a convenience sample of 19 participants representing a range of roles and associated experience. Colleagues provided us with mediated initial contacts, who then invited other colleagues of theirs to participate. One focus group comprised nine ear, nose, and throat attending and resident surgeons; the other involved five attending orthopedic surgeons. The five individual interviewees were a pediatric orthopedist who directs the surgical residency program at an academic medical center; a medical assistant for a busy multi-physician orthopedic practice who interacted daily with device reps; and two former and one active medical device reps.

Focus groups were conducted in departmental meeting rooms during times normally set-aside for morning or noon conference, with modest refreshments provided. Interviews were carried out in department meeting rooms or available classrooms at the medical school. All interviews and focus groups were conducted in person by the authors, and were audio recorded and transcribed near-verbatim, omitting only “placeholder” locutions (“uh,” “um,” and the like). Field notes were hand-annotated and accessible to interviewees. Respondent anonymity was preserved by removal of identifiers from final transcripts. No raw data or subject identifiers were available to anyone but the research team.

We formulated a preliminary topic list, broadly based on issues identified in the considerable literature on pharmaceutical industry-physician relationships and their known or potential influences on physician choice, prescribing patterns, treatment decision-making and unconscious biases, to serve as our initial focus group and interview guide. As is customary in exploratory ethnographic research, topics were added to the guide as they were introduced by participants; retained if they prompted vigorous discussion or strong statements of opinion; and deleted from the list if participants did not respond with interest or confirmation of their importance.

Analysis of the qualitative material followed established ethnographic conventions of narrative content analysis, in which statements of belief, opinion, or putative fact are taken as the units of analysis, and identified as narrative themes. Illustrative quotes explaining individual or collective positions on topics and themes were extracted from interviews and focus groups to present a range of opinions and rationales surrounding specific themes. Quotes were selected primarily on the basis of clarity of statement or fluency of expression with respect to these particular points; omissions of redundancies and placeholder locutions are indicated in the quotes by ellipses (…). In accordance with our guarantee of confidentiality to participants, direct quotes cited herein are attributed only to profession: Ear, Nose, and Throat Surgeon (ENT), Orthopedic Surgeon (Ortho), Surgical Residency Program Director (RPD), Medical Assistant (MA), and Device Rep (DR 1, 2, or 3).

## Results

### Standard Operating Procedure: The Device Rep Is Almost Always There

A device rep is generally a fixture in the operating room during orthopedic procedures, preceding the surgeon into the OR to ensure that all needed equipment is at the ready.

“Nowadays, a [device] rep is in the OR for every case that you’re using his or her stuff.” [Ortho]

“I worked in joint construction [where] it’s accepted, it’s common practice… that the device rep be there for the entire surgery.” [DR1]

“[The orthopedic surgeons’] medical assistants had to send [the] surgical schedules to their device reps to make sure [they] were always there for the surgery.” [MA]

Planned exceptions to this rule include simple procedures that do not involve implants and cases where a particular surgeon and device rep concur there is no need for the rep to be present.

“Pediatric orthopedic surgeries, exclusive of the spine surgeries, rarely involve the use of implants… [those] we use most commonly we use on a daily basis. [The rest is] simple wires, simple pins, simple screws… so there’s really no need to have a rep in the OR.” [RPD]

“I’ve had reps tell me, ‘I know you have a case, but I can’t be there because I have to be with another surgeon who needs me more than you need me.’” [Ortho]

“If it was a fairly simple system to use, I would ask the surgeon, ‘Do you even need me there for that?’ And if they’re reasonable people, they usually say, ‘You know, this is easy enough…you don’t need to come.’ So I would just have the stuff sent in.” (DR2)

### Role of the Device Rep

From all accounts, device reps perform a myriad of tasks related to their OR assignments. Our interviewees reported attending at least one surgery daily and having to be available, at least by phone, 24 hours a day, seven days a week.

The reps are responsible for ensuring that all the instruments and components needed for each surgery on their schedule are on hand and ready for use. That may entail assessing and accessing hospital stocks, as well as bringing their own implant systems. They also anticipate the need for alternative sizes, instruments, and components, and they bring these additional items with them. One rep reported helping hospital personnel sterilize and rewrap instruments after each surgery.

The orthopedists estimated that they used about 100 implantable devices, not including the other tools used during any given surgery. Device Rep 1 estimated that for a total knee replacement, there are “typically eight trays of instruments, each with between 30 and 60 pieces of metal,” with all of which he was singularly familiar.

“This is one of the reasons why, currently, you need a rep in the operating room. . . . Who’s to say who else will know when we get to surgery in the morning whether you're opening the right implants, or whether you're opening the right trays?…[I]t's useful to have somebody else in the room whose specific job is to have that information on hand always.” [DR1]

“When the doctor comes into the room, [I can say], ‘We're ready to go with the instrumentation.’ That's one less thing he has to think about or worry about. And he has to be able to trust that I can say that with confidence.” [DR3]

The device rep wears scrubs and authorizing badges in the OR, but does not scrub in and is not allowed to touch anything. He* [our three interviewees were males, as are most device reps][16] uses a laser pointer to indicate to the scrub tech(s) the items to be handed to the surgeon, in the appropriate order.

“I like to say that the surgeon never has to put his hand out and ask for a scalpel; he just puts his hand out and gets what he needs.” [DR3]

Throughout the surgery, the device rep is there to guide the scrub techs as needed, to answer any questions the surgeon may have, and to suggest or provide alternatives should a problem arise.

The extent of direct exchanges with the surgeon during the course of the surgery varies considerably. The rep takes his cue from the surgeon in a speak-when-spoken-to-manner, answering any questions posed to him but otherwise maintaining silence and a fit distance from the surgeon’s activity. To do otherwise, said one ENT, could get him removed from the OR and not invited back.

“There was a big range of what you did. There were surgeons where, literally, all you did was open a box. They didn't need your help; they didn't want your help. They knew the sizing perfectly. . . . And there [were] other surgeons who would…ask for step-by-step instructions at times.” [DR1]

Their auxiliary role in the surgical suite notwithstanding, each device rep recounted instances of having saved the day by dint of having a more thorough familiarity with the equipment and/or possible maneuvers than the surgeon had.

“I have seen a doctor fracture a trochanter while putting in a hip replacement and look up at me and say, "Hey, do you happen to have your cables?" He had not ordered cables. . . . [But] we made it standard practice to always bring it with us. So this is one of those situations where I get to be the hero. He's never fractured a femur before. . . . So, I run out and I get [the cables, which are wrapped and sterile and outside the room] and I hand it to the circulating nurse, who will open the tray and put it on the table, and [the surgeon says], ‘Okay, walk me through this, I don't know how to do this.’ I'm the person in the room with the most knowledge about that device at this point. I know how to thread the wire through the jig. I know how it goes into the plate. I know how much you have to tighten it…” [DR1]

## Qualities of Device Reps that Surgeons Appreciate: A Delicate Balance

Surgeons and device reps alike named reliability the most valued quality of a device rep: always being available and in the OR as planned, bringing all the needed equipment, knowing the equipment, and being able to troubleshoot. The complementary quality, expressed by many of the surgeons, was knowing their place.

“Humble, know their restrictions, recognize their limitations,…experienced, know their products.” [RPD]

“…the girl, I think she’s really good…because she is quiet, she knows what she’s doing; she does what you want her to do. If you’re frustrated with a device, she comes in, she does her magic, she fixes it, and she shows you how to fix it next time.” [ENT]

“We just want them to have the information; if something doesn’t work, to fix it.” [ENT]

“Availability is what really makes a good rep. To be able to answer the questions you want answered but not to come and pester you.” [ENT]

“I want my inventory to be there when I use it, and I want my rep to be responsive…right now I have three different reps. . . . They do everything for me. I’m comfortable with them.” [Ortho]

Surgeons expressed mixed messages about their regard for device reps. They tended to value those individuals they’d chosen to associate with, crediting them with improving the efficiency of the OR team. Praise was more sparing from ENTs than from orthopedists, who described “their” reps as being like reliable sidekicks.

“The rep is another set of eyes. They know the system and the trays—and when you’re not worried, that helps you focus on the patient more because you know what’s coming into your hand is the right next step.” [Ortho]

“If there are ten different screw drivers, they can say, ‘Listen, use this screw driver first.’ And if you’re struggling to get something in, they can say, ‘You can try over there, there’s one that’s angled the other way’…a good rep can add value to the procedure.” [Ortho]

“When the rep isn’t there, there are times when I’ve had to walk around the table and pick out the instrument myself and say, ‘No, this is the spreader. This is the compressor. This is the device, the tool that I need.’ That’s not good.” [Ortho]

“…reps are sometimes helpful…if we want a certain screw size or a certain type of screw plate, [the rep] can point directly and say, ‘This is what the surgeon wants.’ It’s helpful when I don’t have to go to the back table to find the right instrument.” [ENT]

“They never say anything like, ‘this is where screw A is better than screw B.’ They know better than that. But if we ask them, ‘do you think this size will fit better than this?’ they’ll say, ‘well, I’ve seen a lot of surgeons using this one and it seems to be working for them.’” [ENT]

In general, ENTs tended to talk about device reps in the aggregate as pests.

“They will notice as soon as you graduate. . . . They’re there [at meetings] to shop for surgeons. It’s really easy for them because all our information is in the database;…they have a bar code scanner and they just scan our badge…and they actually ask: ‘What is your role? Are you the one that decides, are you the one that suggests [what is purchased]?’” [ENT]

“Some reps are following surgeons around [at the hospital], sitting in the doctors’ lounge, hanging out. . . . They try to pester us: ‘Hey, what’s going on?’” [ENT]

“…there was actually this one pushy rep who came to our clinic and wanted us to use a new device for sinus surgery, a balloon. He somehow got the names of patients…He e-mailed me, ‘Can I come in for these cases?’…We refused to use his product.” [ENT]

“There are some things that are good about having a rep around in the OR if you need a part and if you don’t know what it is or where it is. They can help with that kind of stuff, but if they ever try to tell me how to do something–Done.” [ENT]

### Wooing the Surgeon: The Device Reps’ View

Device reps tended to view surgeons as potential clients to be cultivated. They strived to ascertain the kind of guidance that would be appreciated or rejected and how best to maximize the comfort level of the targeted surgeon.

“I have to understand what a surgeon is doing, what he is using and all the circumstances that are involved with his practice and then I kind of target the things that I can address with either my products or my personal skills or something that the company can offer. . . . [A]s you become more confident you are more suited to approach surgeons and ask them to use your products, if for no other reason than you're ready and willing to be able to help them. And that doesn't necessarily mean that the devices are better than other devices but once again that the representation is there. I really put a lot of weight on my personal representation.” [DR3]

“I was told explicitly by very successful salesmen, you have to be thinking about what kind of doctor your person is. . . . For example, it is not good to show up driving a car that is more expensive than the car that your surgeon drives. . . . If you develop that good relationship and he likes you and he feels that there’s no difference between my plate and their plate, my knee and their knee, he’ll just go with the guy he likes more, the guy he trusts more. So if you have everything your competitor has, if your portfolio matches, then he never has to use the other guy and you convert all of his business. That’s the goal.” [DR1]

The reps also had their share of critical comments about the surgeons they’d worked with, recalling instances in the OR of surliness or impatience that did not benefit the patient on the table.

“Some surgeons are just impossible to deal with. And the funny thing is those are usually the ones that are feeling an inadequacy about what they’re doing. . . . It’s the ones who are habitually not ready for prime time and going into surgeries without a plan that really, really upset me.” [DR3]

One rep said he eschewed surgeons who seek financial relationships with the manufacturer; another faulted surgeons who insist on using high-end equipment for all cases, regardless of the actual need.

“There’s also surgeons out there that aren’t interested in relationships with companies where they can’t have a financial incentive for representing a particular company. . . . I’ve found it best to work with the guys that don’t do that.” [DR 3]

“The orthopedic and development landscape is a fairly tight network. . . . As surgeons became more savvy and sophisticated…they started demanding more features, more options for the devices. . . . That obviously gives a device company a substantial opportunity to make a financial profit…so that $300 plate is closer to $2000-$2500…and the problem I’ve seen is that you don’t always need to drive a Ferrari; most of the time the Toyota Corolla will be fine…[but] once surgeons get used to using a Ferrari, they always want to drive the Ferrari. . . . The surgeon isn’t paying for it. The hospital is. . . . It starts spiraling out of control. What I see as a big issue is surgeons being able to say, ‘No. For ninety percent of my fractures the $300 device will be fine.’” [DR2]

He did, however, acknowledge the role of the device rep in the overutilization of higher priced equipment.

“When I worked as a sales rep, I wasn't paid a salary. It was all commission. I was paid twelve and a half percent of every dollar that went in. . . . And doctors kind of know that…they understand that the rep is just trying to earn a living. They feel somewhat obligated to use the most expensive device because they obviously called you in for it.” [DR2]

### The Device Rep and the Office Staff: A Member of the Family

The medical assistant interviewee portrayed the actions of device reps in the surgeon’s office. In the practice where she worked, each orthopedist had both an MA and a device rep. During the two years she was there, all the device reps were male and all the MAs were female. Occasions for after-work happy hours or dinners were common.

“The device reps were very nice, affable…in the office almost daily. We enjoyed their presence. . . . They came in to get X-rays. . . . They would come by and just chat with us…They’re very much a part of the team…almost part of our family.” [MA]

The interactions between the surgeons and the device reps were also friendly.

“Each [surgeon] had their own device rep…custom tailored to their personality, styles, and whichever company they liked. . .” [MA]

Her own surgeon, who performed between five and eight knee replacements each week, routinely asked the device rep to stop by to discuss what would be needed in the OR.

“When the rep would stop by, it was, ‘Hey, buddy, how’s life?’” [MA]

“When you’re in the OR for someone five hours a day, [two days every week], I think you’re going to be close with them.” [MA]

A key item in the medical assistants’ work orders was to ensure that the device rep knew the surgeon’s schedule.

“[My] device rep always knew when the surgical cases were. I didn’t have to remind him. Other medical assistants had to send schedules to their device rep. . . . I remember when one of my co-workers got in trouble because she forgot to plan with the device rep to be there for the surgery.” [MA]

### The Device Reps’ Bottom Line

Device reps earn as commission a percentage of every sale they make. The moment the sterile packaging is opened in the OR—both items previously selected and those that may be enlisted on the spot during the surgery—the rep earns the commission. The more expensive the equipment, the bigger the commission. This fact of business life may influence not only the products the rep recommends for a particular surgical procedure but also whether or not he will be enthusiastic about attending the surgery at all.

“The big cases for which I more or less had to be present every time were external fixation cases. And the reason for that was because those were high dollar…[averaging] about $5,000…[so] I’m looking at close to $600 for a case that’s only going to take an hour. . . . You’re always happy to do ex-fixes. . . . I had a substantial [number] of hospitals where I did ex-fixes. I probably did close to $40- or $50,000 of external fixations a month in some instances. So it was a big money maker for me…” [DR2]

Conversely, he would calculate whether the time and money expended in traveling to a hospital and attending a procedure would exceed the commission he would earn from sale of the product. A losing proposition could lead to a talk with the surgeon about not needing a device rep to be in the OR.

Another rep grappled with the impact on patient care of his promoting newer, more expensive products that would increase his commission.

“[So I tell the surgeon], ‘Just so you know, I noticed the patient was young, so we also ordered ceramic heads. The coefficient of friction is lower [and] should make the part last longer.’…I don’t know if that’s true or not. . . . That’s what we were taught to say. . . . [The ceramic] costs three times as much as the other. . . . So you can see how I have a direct incentive to get a surgeon to use something more expensive. . . .There was never a situation when I thought, ‘I know this is inferior and I’m going to sell it anyway.’ [It was more like] ‘I don’t think this is any better, but I know it’s more expensive, so I’m going to sell it.” [DR1]

He was also ill at ease with the lack of data supporting new product claims and the impact on health care costs.

“I often felt like I’m driving up the costs of the health care system. . . .We used to sell an implant that has 99% survivorship at 15 years, which is great, right? We were told to not ever market it to anybody. . . . If a doctor asked for it by name, we would give it to him. We want to market the newer, the better technology. I’m not certain I ever thought the newer technology was better. There certainly wasn’t data on it. . . . I was uncomfortable with those sorts of things.” [DR1]

The medical assistant lamented her own role as a surrogate for the device rep, who could not speak to the patients on his own. Her assignment from him was to champion a new, costlier custom-made knee replacement system over an implant that was already available in ten different sizes. There was no concrete evidence that the custom-made knee conferred an advantage in terms of faster recovery time or longer product life, she said, and its use required patients to obtain a preoperative MRI four weeks before the surgery.

“The device rep would tell me, ‘Hey, remember to keep getting people to use the new knee.’ He was persistent, but he’s nice, I like him, and I said okay. . . . I was more of a puppet in that relationship because he would hand me all the material to put in the surgery packet. . . . He’d be my go-to resource for what to tell the patient if they had questions.” [MA]

### Costs and Device Selection Dynamics

There was a consensus among the surgeons that competing devices differ mainly in their respective costs and the quality of the services afforded by the device reps who sell them, two factors cited as major influences in their equipment choices.

“I try to minimize the number of device reps and companies that I work with because I need to be efficient. . . . I use [the reps] because they give me good service. . . . I think that the products are all fairly similar.” [Ortho]

The overarching influence on device selection today, interviewees repeatedly noted, is the economy: the advent of hospital systems and cost-cutting contract pricing has restrained the ability of both surgeons and vendors to add new products to a hospital’s inventory.

“I've been at this for forty years; clearly it was very surgeon driven. [But] as the economy has dramatically changed, and evolved hospital systems…are looking at the power of purchasing, we have said, ‘well, we're comfortable choosing from these two or three vendors, who can supply our needs. And you contracting people can go deal with those vendors.’ And obviously [a large hospital system has] a certain leverage in getting price controls and constraints in service and everything else.” [RPTD]

“We can’t just call the rep and say, ‘show up tomorrow with this new implant.’ [The hospital is] not going to let you do that.” [Ortho]

Vendors prepare elaborate cost-effectiveness reports to present to hospital formulary committees, noted several interviewees, one of whom was on his hospital’s formulary committee. The medical assistant recalled frequent visits by a device rep to see not only his own surgeon but also the group practice member who happened to be on his hospital’s formulary committee.

While surgeons are constrained by hospital contracts, they can still be successful advocates for introducing desired new items into the hospital inventory.

“[What] has the most influence over what I use is the hospital purchasing department. Whatever they’ve contracted to have in the building is what I use. . . . [But] if I want to go through a process to bring in somebody else who has different stuff that I really think is going to help the patient and I really think is going to be cost effective, then I do that.” [ENT]

One ENT interviewee described at length his successful campaign to switch to a different post-laryngectomy voice prosthesis that colleagues elsewhere had recommended as better for patients and more cost-effective. On the other hand, ENTs also described situations where they’d been forced to accept unwanted new technology at the hand of “company” maneuvering.

“Some companies are sneaky. If a device works fine, they do a little tweak and they’ll charge more for the newer device, but then they’ll discontinue the one that worked just fine and then you’re forced to buy the new one.” [ENT]

“I thought we were going to keep [an old system] in the hospital because I wanted it for backup and they said no…it’s all under contract.” [ENT]

A more acceptable maneuver was the free trial.

“A device rep may introduce a new product into a surgeon’s practice [by letting him] try it out free of charge…they gave us free wands and there was no charge to the hospital. We did maybe 10 or 20, and we talked to our patients. Anecdotally, they didn’t have any increased complications; they had less narcotic dependency and were happier with it…and we started using it.” [ENT]

### When Use Outpaces Proof

Word of mouth goes a long way in generating surgeon enthusiasm for an innovation, especially early on—largely because there are few evidence-based sources to assess outcomes. Hospitals typically do not track device performance but some surgeons, surgical practices, or departments conduct their own studies.

“As far as device success rate, the hospital doesn’t keep track of that. . . . As physicians and surgeons we [could] track [that information] down…if we really wanted to know that.” [ENT]

“[There are] people who are doing specific outcomes-based studies. [But] it’s not just something that happens automatically; the surgeon or investigator has to be proactive.” [Ortho]

“There are no formal tracking methods for institutions or hospitals. . . . But in Scandinavian countries, they have a joint registry where every patient is…followed over time until they die.” [Ortho]

One orthopedist expressed confidence in the FDA’s 510(k) process for expedited approval of devices with “just a little design change but materially similar to a product already on the market.” But another recalled an instance when proof of “equivalence” was controversial and did not guarantee an unblemished performance on the market.

“I’m not an expert on the FDA, but [equivalence] has come up in spine surgery implants with pedicle screws. Twenty years ago, there was a huge debate over whether this was a new device. . . . It’s really revolutionized…spine fusion surgery, but there are certainly some dangers involved, so when it was popularized in the nineties it was very controversial. There were class action lawsuits and a lot of negative publicity.” [Ortho]

Off-label uses of devices was also mentioned. One ENT surgeon cited an instance when off-label use so alarmed the manufacturer that it issued a warning against that use.

“With a [purely] mechanical device [say a knife, as “opposed to a CO2 laser”], the surgeon doesn’t care if it’s off-label. . . . [But] you have examples of off-label uses that actually cause problems to the point that the company took action, like recently in robotics…[with a device] proven in cosmetics surgery…but not approved for thyroid surgery. . . . I guess something must have happened [because] suddenly the company issues a statement…loud and clear…that it is not approved for thyroidectomy. So I’m assuming they saw some kind of complications and didn’t want to be liable for it.” [ENT]

### Taking Industry’s Word for It?

The device rep often serves as the conduit of information about new products, and the device manufacturers provide hands-on training courses for surgeons and residents. In general, our surgeon participants regarded industry-provided education and training as desirable, suspect, and a necessary evil, in varying measures.

The residency program director nostalgically recalled bygone weekly gatherings at lunchtime, when sandwiches and new products were laid out on a table for all to digest.

“The problem currently in my view is that we're throwing the baby out with the bath water. . . . This department once a week would have a lunch,…vendor sponsored…sandwiches for about 20 people. . . . Personally, for myself and for our residents, I found that to be an educational opportunity. We got the chance to look at new products,…to hold [them] and to hear the spiel. None of us is so naive as to [think] that these are not salesmen and they are not selling stuff…[and]…if people truly believe that we can be bought with a turkey sandwich, then I think the absurdity of that speaks for itself. . . . I think in today's day and age…we have gone so far to the opposite extreme that it makes it really difficult to even see what's new.” [RPD]

The program director was unconcerned about sending his residents to industry-sponsored events. He felt he exercised sufficient control over the content of such sessions to counter any bias that otherwise might be introduced.

“We allow our residents to go to vendor-sponsored courses. I, as a program director,…look at the program itself before we let our residents go. . . . I’m in the position of telling the vendor what I want taught at these courses. . . . It's an [educational] opportunity for these residents. And because they go to three or four [different] courses in the course of a couple of years, there's no one vendor who has a corner on the market.” [RPD]

Factors that he felt protected his training relationship with vendors from bias included.

“I don't gain any consulting money. I feel like I'm a relatively honest…judge in terms of looking at…the science. . . . [M]y job is to make sure these residents are educated on these devices because they are the best; they have withstood the test of time.” [RPD]

Online learning modules, he added, are another tool of vendor participation in resident training, for which the residents get credit that can be exchanged for “legitimate textbooks or journals.”

The orthopedic focus group discussed at length whether and with what boundaries industry-sponsored training could be attended, noting that they themselves typically ignored the unsolicited suggestions of device reps to avail themselves of training in a new technique, but called upon the rep when the time came that they considered the technique now worth learning.

Two surgeons recounted contacting their device reps to find out when and where courses might be available, on a new spine fusion technique in one case and the anterior approach to hip replacement in the other. In the former, training utilizing a cadaver was being offered on an upcoming Saturday just a short drive away from the surgeon’s base of operations. In the latter, the available training involved a trip to another state and the company paid for the airfare. That situation, in hindsight, he felt was “technically unethical. . . . I should have paid for the plane ticket myself.”

Surgeons appreciated vendor-sponsored meetings as sources of early information on new products, but their enthusiasm was tempered by their assumption of bias on the part of the paid speakers.

“…you hear about [device performance] at meetings, and it’s usually industry-driven. Usually they have a consultant that’s actually using the product and introduces some of the early outcomes. . . . You obviously have to look at that with some caution because they’re an employee of the company.” [Ortho]

“It’s biased information, but it’s all the new stuff they’re working on.” [Ortho]

“Let’s face it. If you go to a course by a vendor, it’s a sales course.” [RPD]

Members of the orthopedic group rued the loss of some of the educational opportunities in the “new world of ‘contact-with-industry-is…bad-because-they’re-all-just-marketers.’”

“You can get information about a new cholesterol-lowering drug in the literature or at a society meeting…[but] to learn how to do a new spine procedure you need industry to teach you how to use this new equipment they’ve developed.” [Ortho]

In a similar vein, surgeons drew a distinction between drug reps and device reps, the former

“…coming in and trying to buy you lunch so you’ll write prescriptions for their pills, versus technical reps who help facilitate procedures.” [Ortho]

“…you don’t have to go to a weekend course to write a prescription, but you do have to go to a weekend course to learn how to do a [procedure].” [Ortho]

Although surgeons acknowledged that the ideal teaching situation would be bias- free, several noted the unrivaled ability of the device industry to bankroll major conferences, and the reluctance of medical institutions to fund training.

“[A major company] holds a conference once or twice a year; they pay the speakers $5,000, and they’re very good; they’ll provide the means necessary for the residents to travel. . . . They actually invest a lot of money on education,…but it’s obviously one product. So if you’re educated by them, you get biased toward using their products.” [ENT]

“Here at the university we get paid zero dollars for educational activities. . . . Conferences cost money…and we have to pay [the university] a lot of money for [them] to provide CME. Imagine that. We are full-time university faculty and we are creating an educational activity with the name of the university…and nobody will provide us with any money. Neither the hospital nor the university. So we charge the participants some fee…and for the rest we go to different companies and device reps and drug reps to be able to make education happen.” [ENT]

“The problem is at the level of the entire economic system. . . . There are two ways to fund the health system. [P]ay more taxes and use that money…as a society to invest in education…[or] privatize everything;…let the doctors, hospitals, educators go after [the companies] and beg them to help their education, help the universities [with] research funding. . . . We have decided we’re going to empower the industry. . . . That’s the bottom line.” [ENT]

### Are Device Reps in the OR Here To Stay?

In separate interviews, two device reps independently stated that the only reason they had their jobs was because surgeons weren’t fully doing theirs.

“[Surgeons] handed over the reins to us: to salesmen. So if we'd never been there in the first place, or if we left, they'd have to restructure and eventually they'd get much better. . . . I needed to be there…because either the staff or the surgeon hadn’t bothered to be trained. . . . It wasn’t always this way. It used to just be you dropped [the devices] off and you left. But one company said one day, ‘Hey, if you buy it from me, I’ll be there and help you put it in because it’s complex.’ And they started doing really well…outcompeting everybody. So every company started offering the same service.” [DR1]

“I think surgeons need to take more responsibility. . . . The minute they're asking for a device they should be an expert on it. . . . Going through medical school and going through all the training you go through…just to have another person come in the room and tell you what to do? I have a problem with that. . . . I think the surgeons should be good enough to make their own decisions. They should be able to do the case on their own.” [DR2]

This sentiment was echoed by the ENT surgeons.

“It seems like we sometimes rely a lot on the vendors so far as how to do a certain technique, or what to do in a situation, which irks me because I’m basically asking a rep ‘how do I do this?’ during the procedure. . . . As a physician, you’re the one who should know what to do, you should have the knowledge. . . . You shouldn’t be relying on the rep to help you do the surgery at all.” [ENT]

“I think most of the time we shouldn’t need them. The problem is that…after a while, that becomes the norm so nobody makes an effort to learn things properly…it doesn’t need to be like that. . . . [V]ery good nurses and very good techs [could] troubleshoot problems. So in a way, the hospital might be trying not to train their own people just because they have access to device reps. . . . In my opinion, some of our people are just not very good because of this.” [ENT]

On the subject of “need” for a device rep in the OR, the device rep interviewees (all trained in orthopedic devices) had varying perspectives. The currently active rep was unequivocal.

“Positively, without a doubt. I think it would be a mistake to go into a surgery without the representative being there.” [DR3]

Two former reps, currently in medical school and considering becoming orthopedic surgeons, felt differently.

“I believe that salesmen influence…treatment options for surgeons. And I don't want a [salesman] making the decision. I want an educated doctor making the decision. . . . In order for me to be an effective doctor I think it's important for me to limit my exposure to things that are going to sway my opinion that aren't based in medical fact. And I think that number one on a list of things that sway your opinion…that aren't based in medical fact comes from industry and from particular salesmen.” [DR1]

“…I know of a hospital that doesn't allow any reps in the OR. None at all. The hospital staff is very comfortable using the total hip and knee systems in house. . . . [T]he hospital took it on themselves to train their employees. . . . [I]f there's a problem, [the surgeons] figure it out. They train themselves, they become self-sufficient. There's no middle man. Reps are middle men, right?…[But] do hospitals want to take that responsibility?” [DR2]

DR1 suggested that if surgeons curbed their enthusiasm for new technology (“don’t be an early adopter”) the need for device reps would certainly shrink.

Device reps also differed in their perspectives as to whether the presence of reps in the OR creates an inherent conflict of interest. One thought not.

“I just want everything to go well, to go fast, and to go efficiently. . . . My whole [aim]…is to get the patient into the room and out of the room. Make sure the instruments are ready,…This reduces the amount of time that the patient is on the table, which in my opinion is the most critical factor. . . . If we get the patient out faster it's beneficial for everyone: cuts OR time…(and) if there's more time for more surgeries, I may have more time for another opportunity to make money. The doctor's schedule is freer…[and there’s] lower infection rates and less morbidity the less time that [patients are] under. So it really is about patient care, but there's a lot of other benefits that go along with it.” [DR3]

But another disagreed.

“The way I look at it, once I become a surgeon…I've crossed a line. My interests are no longer in alignment with the company’s bottom line. . . . In fact, it's going to be the exact opposite. I'm interested in how I can obtain services and products at the lowest cost and pass that savings down to my institution and my patients. . . . Because remember, the minute a rep sits in the room…their goals are not in line with the surgeons’ or the patients’. Their goals are in line with the company’s interests.” [DR2]

## Discussion

Surgeons and device reps enjoy close working relationships. While surgeons appear to see themselves as indisputably in charge, device reps work hard to make themselves unobtrusively indispensable in an effort to maintain their influence, and to imbue the products they provide with personalized services that foster a surgeon’s loyalty to the reps personally, and by extension to their products and company. Surgeons who were uncomfortable with some roles of reps nevertheless defended their own relationships with their personally selected reps. Training opportunities provided by companies are also viewed as a helpful service; even physicians who were uncomfortable with this situation noted, with frustration, that they had found no viable alternative to industry-funded courses for receiving continuing education or providing practical experience to residents.

While it is clear that surgeons benefit from relationships with device reps, benefits to patients arising from that relationship are ambiguous. Impact on patient care must be viewed in the context of:

- the FDA approval process, which forgoes clinical trial requirements for devices deemed substantially equivalent to already marketed products.
- the practice among manufacturers of crafting minor modifications to their implantable products, which arrive relatively quickly on the market accompanied by as yet unproved claims of superior clinical performance as well as higher prices.
- the financial interest of the parent company and individual device reps in selling the most expensive implants, which may not necessarily be the best choice for individual patients.
- the paucity of independent peer-reviewed outcomes data to fortify a choice to switch to the newer model.
- the dominance of company representatives and surgeon consultants on their payrolls in the dissemination of early information on their new products and training sessions for surgeons.
- the lag time between widespread adoption of a new technology and the emergence of peer-reviewed evidence-based reports of its benefit or, conversely, its unfitness.

The perception among reps and some surgeons that reps benefited patient care indirectly, by increasing efficiency and mitigating deficiencies among operating room personnel (including, in some cases, the surgeons themselves), raises disturbing questions about acceptable levels of competence among essential OR personnel. Device reps are highly trained on their company’s products, but are not trained on competing products; thus, they cannot provide an unbiased assessment of competing products. The importance of having unbiased comparisons and assessments of surgically implanted devices to foster evidence-based practice cannot be overstated–especially as medical devices have both faster development times to come to market and far less published data on therapeutic superiority or equivalence than do pharmaceutical products.[4]

Perhaps salaried individuals performing the usual tasks of today’s device reps—but trained on a variety of devices, with no financial incentive to promote one over the other—ought to be employed by hospitals or health care systems. In this way, the value they surely impart could be preserved, comparative performance data gathered systematically, and undue industry influence curtailed.

A program for “surgeon assistants”—not unlike those for physician assistants, but incorporating the sort of technical training device reps currently receive—could produce that useful tier of health care providers to serve as “another set of eyes” in the OR without risking compromise by industry profit motives, or the competing priority of reps’ needing to safeguard their personal bottom lines. Moreover, early utilization of the latest innovations would lose some of its cachet.

At least as important would be the systematic documentation of short- and long-term intra- and postoperative experiences and clinical outcomes among patients receiving implants. Rather than emphasizing training in use of new technologies—with the concomitant implication that “new” is preferable to “old”—it would better serve the public health to compare new technology to old before adopting it.

This was an exploratory study with modest funding, which limited its scope to a small convenience sample size (n = 19). Although we were able to include differing roles among our respondents (surgeons, device reps, medical assistants), our sample touches on only two surgical specialties of the many that use implantable devices. Qualitative methods, which are ideally suited to discovery and investigation of opinion, belief, values, rationales and the like, do not address such matters as broad generalizability across an entire field, or predict how commonly their narrative themes might be discovered in differing regions of the country, other surgical specialties, sizes and types of hospitals, etc. Our study does not address sociological factors such as rank and its influences in focus groups, nor other forms of interpersonal and group dynamics that may influence responses and conversational exchanges in groups. We did not attempt to stratify focus groups along any demographic variables such as gender, age, years in practice, title, or relative status, which may influence group interactional patterns and responses. We selected narrative content analysis as our analytic approach; this is only one of several valid approaches to analysis of a corpus of narrative data, such as turn-taking and other interactional factors, performative elements of speech acts, or narrative structure, to name a few.[17]

## Conclusion

The issues and potential pitfalls of excessive industry influence in medical care and physicians’ treatment decision making are at least as urgent for implantable medical devices as they are for pharmaceuticals and prescribing practices, but to date they are far less well studied, debated, or systematically subjected to comparative effectiveness trials. We call upon professional societies, hospital systems, individual hospitals, collaborative private practices, and regulatory bodies to develop real-time, workable methods to systematically collect, analyze, and disseminate data on implant surgeries and their short- and long-term outcomes to the professions and the public.

Finally, academic medical centers and health care systems must develop means to provide neutral financial support for educational conferences and device training for their surgeons and trainees.
